# Alterations of cerebral intrinsic activity in first-episode, drug-naive patients with major depressive disorder

**DOI:** 10.1192/bjo.2025.65

**Published:** 2025-06-27

**Authors:** Xiaoxiao Hu, Xinyu Hu, Hailong Li, Lianqing Zhang, Lu Lu, Xuan Bu, Shi Tang, Qiyong Gong, Xiaoqi Huang

**Affiliations:** Department of Radiology, The First Affiliated Hospital, Sun Yat-Sen University, Guangzhou, China; Department of Radiology and Huaxi MR Research Center (HMRRC), Functional and Molecular Imaging Key Laboratory of Sichuan Province, West China Hospital, Sichuan University, Chengdu, China; Research Unit of Psycho-radiology, Chinese Academy of Medical Sciences, Chengdu, China

**Keywords:** Major depressive disorder, first episode, amplitude of low-frequency fluctuation, resting-state functional MRI, drug-naive

## Abstract

**Background:**

Major depressive disorder (MDD) is a leading cause of disability worldwide. Investigating early-stage alterations in cerebral intrinsic activity among drug-naive patients may enhance our understanding of MDD’s neurobiological mechanisms and contribute to early diagnosis and intervention.

**Aims:**

To examine alterations in the amplitude of low-frequency fluctuation (ALFF) in first-episode, drug-naive MDD individuals and explore associations between ALFF changes and clinical parameters, including depression severity and illness duration.

**Method:**

A total of 30 first-episode, drug-naive MDD individuals (mean illness duration 14 weeks) and 52 healthy controls were included in this study. Resting-state functional magnetic resonance imaging was used to obtain whole-brain ALFF measurements. Voxel-based ALFF maps were compared between MDD and healthy control groups using a two-sample *t*-test. Simple regression analysis was performed to assess associations between ALFF and clinical measures, including Hamilton Rating Scale for Depression (HAMD) scores and illness duration.

**Results:**

MDD individuals exhibited significantly increased ALFF in the dorsal anterior cingulate cortex and vermal subregion V3 of the cerebellum. Additionally, ALFF in the right dorsolateral prefrontal cortex was negatively correlated with HAMD scores (*r* = –0.591, *P* < 0.001). However, no significant association was found between ALFF and illness duration.

**Conclusions:**

This study demonstrates early-stage ALFF alterations in drug-naive MDD patients, particularly in brain regions implicated in cognitive and emotional regulation. These findings suggest potential neuroimaging biomarkers for the early diagnosis and intervention of MDD.

Recent global analyses have highlighted the substantial burden of major depressive disorder (MDD), with a 59% increase in incident cases from 1990 to 2019 despite a decline in age-standardised rates.^1^ Women and older adults, particularly those in low-sociodemographic index regions, bear a disproportionate burden of MDD. The intricate interplay of risk factors, including intimate partner violence and childhood trauma, underscores the urgent need for targeted and personalised interventions.^1^ Moreover, the strong comorbidity between MDD and physical diseases such as cardiovascular disorders and diabetes reveals the involvement of shared biological mechanisms, including systemic inflammation and dysregulation of the hypothalamic–pituitary–adrenal (HPA) axis, which further compound the disease burden.^2^ Gaining a deeper understanding of the neurobiological underpinnings of MDD is essential for advancing treatment strategies and improving patient outcomes.

## Neuroimaging insights into MDD

Resting-state functional magnetic resonance imaging (rs-fMRI) has emerged as a valuable neuroimaging modality for assessment of spontaneous or intrinsic brain activity in a non-invasive manner, without the need for task-based paradigms. One key rs-fMRI metric, the amplitude of low-frequency fluctuations (ALFF), quantifies the localised intensity of spontaneous fluctuations in blood oxygenation level-dependent (BOLD) signals^3^ and serves as an indicator of intrinsic neural activity within specific brain regions.^4^ Owing to its temporal stability^5^ and test–retest reliability,^6^ ALFF has been proposed as a robust biomarker for identifying abnormalities in cerebral intrinsic activity associated with in psychiatric disorders.

## Confounding effects of medication and illness duration

Prior neuroimaging research has demonstrated that MDD is associated with widespread alterations in ALFF across various brain regions, including the anterior cingulated cortex (ACC),^7,8^ parahippocampal gyrus,^8^ ventral median frontal gyrus^8,9^ and putamen.^8^ The inconsistencies observed among these studies may be attributed to the heterogeneity of the disorder, variations in medication status and differences in illness duration.^10^ Some studies involving MDD individuals receiving antidepressant treatment reported increased ALFF in the parahippocampal gyrus and putamen, along with decreased ALFF in the middle occipital gyrus.^8,9^ Conversely, another study examining drug-naive individuals with MDD found reduced ALFF in the bilateral orbitofrontal cortex and increased ALFF in the bilateral temporal cortices.^11^ These cross-sectional findings suggest that medication status may contribute to the variability observed across studies. Moreover, an increasing body of longitudinal research has shown that antidepressants can normalise the abnormal activation of brain regions in MDD.^12^ For instance, antidepressant treatment has been shown to normalise hypoactivity in the dorsolateral prefrontal cortex (DLPFC) during cognitive tasks in MDD individuals.^13^ Additionally, treatment with escitalopram increased fractional ALFF (fALFF) in the DLPFC, where MDD individuals initially exhibited lower baseline signals compared with healthy controls.^14^ Taken together, these findings suggest that prolonged exposure to antidepressants may serve as a confounding factor influencing neuroimaging results in MDD.

## Rationale for studying drug-naive first-episode patients

Furthermore, research suggests that illness duration and the number of depressive episodes influence the neurobiological alterations associated with MDD.^15^ One recent meta-analysis reported a correlation between ALFF-measured intrinsic brain activity and illness duration,^10^ suggesting that the latter may be a potential confounding factor in neuroimaging studies of MDD. Therefore, to comprehensively characterise alterations in cerebral intrinsic activity in MDD, these limitations must be addressed. Given that the majority of previous studies have focused on chronic MDD patients who had received antidepressant treatment prior to neuroimaging, it is essential to investigate cerebral intrinsic activity in first-episode, drug-naive individuals. Such an approach provides a more reliable means of elucidating the core neurobiological mechanisms underlying MDD, minimising the confounding effects of illness chronicity and medication exposure.^16,17^

## Study objectives and hypotheses

The objective of this study is to utilise voxel-based analysis of the amplitude of the ALFF technique to investigate cerebral intrinsic activity in first-episode, drug-naive individuals with MDD who have a relatively short illness duration. We hypothesise that abnormalities in ALFF activity will be observed in MDD patients compared with healthy controls. We also aim to examine the association between ALFF and clinical parameters such as symptom severity and illness duration. Specifically, we posit the following hypotheses. (a) First-episode, drug-naive MDD individuals will exhibit significant alterations in ALFF compared with healthy controls. (b) Specific brain regions, such as the dorsal anterior cingulate cortex (dACC) and cerebellum, will demonstrate increased ALFF in MDD individuals. This hypothesis is based on prior research indicating that these regions play key roles in cognitive and emotional processing. The dACC is a central component of the cognitive control network and has been shown to exhibit hyperactivation in drug-naive MDD individuals,^18^ while the cerebellum has been implicated in attention and emotional regulation deficits associated with MDD.^7^ (c) The ALFF in certain brain regions, particularly the right DLPFC, will show a negative correlation with depression severity (Hamilton Rating Scale for Depression [HAMD] scores). This aligns with previous findings suggesting that hypoactivity in the DLPFC is associated with greater depressive symptom severity and impaired cognitive function.^16,13^ (d) The observed ALFF changes will provide insights into the early neurobiological alterations associated with MDD, and highlight potential regions involved in cognitive dysfunction. By addressing these hypotheses, this study aims to enhance the understanding of the early neurobiological mechanisms underlying MDD and identify potential biomarkers for early diagnosis and intervention. The novelty of this study lies in its focus on first-onset, untreated MDD individuals, thereby eliminating the confounding effects of medication and providing direct evidence of brain activity alterations in the early stages of depression.

## Method

### Participants

In this research, 30 individuals with first-episode, drug-naive MDD and 52 healthy controls participated. These were a part of a large cohort research that looked at severe depressive disorder in Han Chinese people.^20^ The Structured Clinical Interview for DSM-IV Axis I Disorders (SCID) was used by two proficient psychiatrists to verify the diagnosis established for every person. Determining the level of depression included using the 17-item HAMD.^21^ A HAMD total score of >18 on the day of MRI scanning was among inclusion criteria. The following criteria were applied: history of significant medical illness, prior psychiatric treatment, age <18 or >60 years, use of vasoactive medicines, history of alcohol or drug misuse and the presence of psychiatric disorders other than MDD, as defined in DSM-IV. None of those enrolled had received antidepressant treatment before participation in the study. Healthy controls were recruited through public advertisements and matched to the patient group in terms of sociodemographic background. The Structured Clinical Interview for DSM-IV Non-Patient Edition (SCID-NP) was used to confirm the absence of psychiatric disorders in the control group. Additional eligibility criteria for healthy controls included the absence of a personal or family history of psychiatric or neurological disorders. All participants were of Han Chinese ethnicity and right-handed. Written informed consent was obtained from all participants prior to study enrollment. The authors affirm that all procedures conducted in this study adhered to the ethical standards of the relevant national and institutional committees on human research, and complied with the Helsinki Declaration 1975 revised in 2013. Ethical approval for this study was granted by the Ethics Committee of West China Hospital, Sichuan University Hospital.

### MRI data acquisition

A 3.0T magnetic resonance imaging equipment with an eight-channel, phased array head coil (EXCITE, GE Signa, Milwaukee, USA) was used to scan each participant. The following settings were used to create a gradient-echo echo-planar imaging (EPI) sequence that produced rs-fMRI sensitive to variations in BOLD signal levels: field of view 240 × 240 mm^2^; matrix size 64 × 64; flip angle 90°; section thickness 5 mm; intersection gap 0; and voxel size 3.75 × 3.75 × 5.00 mm^3^. Each brain volume comprised 30 axial slices, and each functional imaging session included 200 image volumes preceded by 5 dummy volumes. Each participant utilised foam cushions and soft earplugs to reduce head motion and noise distraction during the rs-fMRI scan. Participants were instructed to relax with their eyes closed, while refraining from falling asleep or engaging in structured or deliberate thought processes. Compliance with these instructions was confirmed immediately after the scanning session. Data were excluded from the study if head movement exceeded 1.5 mm in translation or 1.5° in rotation.

### Data preprocessing

Data Processing Assistant for Resting-State Functional MR Imaging (DPARSF, version 2.3; State Key Laboratory of Cognitive Neuroscience and Learning, Beijing Normal University, Beijing, China; https://www.restfmri.net) was used to preprocess rs-fMRI data.^22^ The following steps were performed: idue to field homogeneity adjustments in the EPI scanning sequence, the initial images may exhibit signal fluctuations. During data processing, the first five whole-brain images from each scan were discarded. The remaining whole-brain images proceeded to the following step of data preprocessing. Slice timing and motion correction: first, slice timing correction was performed using interpolation to compensate for differences caused by varying acquisition times between two-dimensional images of different slices. Next, realignment was conducted using translation and rotation to minimise the effects of head movement between three-dimensional images acquired at different times. Throughout the scanning process, six-parameter rigid-body transformation was applied for head motion correction. Head movement quality control was conducted to ensure the integrity of rs-fMRI data. Participants with head motion exceeding 1.5 mm in translation (*x*, *y* or *z* directions) or 1.5° in rotation (pitch, yaw or roll) were excluded from further analysis. Additionally, framewise displacement was calculated for each time point to assess motion-related variability, and mean framewise displacement across the scan was reported for all participants. Scrubbing was applied to remove high-motion volumes with framewise displacement >0.5 mm, ensuring that only high-quality data were retained for subsequent analysis. These thresholds and procedures were chosen based on established standards for rs-fMRI motion correction, to minimise noise and artefacts. Normalisation: functional images were spatially normalised to the Montreal Neurological Institute (MNI) space using the EPI template. Voxel size was resampled to 3 × 3 × 3 mm³.

### ALFF

Using DPARSF software and a methodology similar to our previous work, ALFF was computed.^23^ The process included the following steps. Time-series transformation: the preprocessed time-series data of each voxel were transformed into the frequency domain using the fast Fourier transform (FFT) algorithm with zero-padding (taper percentage 0) to improve spectral resolution.^24^ The power spectrum was computed as the square of absolute FFT coefficients. Frequency band selection: to isolate low-frequency fluctuations, the power spectrum was averaged across the frequency range of 0.01–0.08 Hz, corresponding to neuronal activity-related signals. High-frequency signals and scanner noise were excluded.

ALFF calculation: the square root of the averaged power spectrum was taken to derive the ALFF value for each voxel:

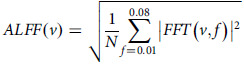




where *v* represents the voxel and *N* is the number of frequency points within the selected range. Normalisation: ALFF values were normalised by dividing each voxel’s ALFF by global mean ALFF across the entire brain, resulting in a dimensionless ratio to control for inter-individual variations:^25^

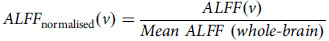




Spatial smoothing: ALFF maps were spatially smoothed using an 8 mm, full-width-at-half-maximum (FWHM) Gaussian kernel to enhance signal-to-noise ratio and reduce spatial variability. Cluster thresholding: a rigorous statistical threshold was applied (*P* < 0.001, family-wise error [FWE] corrected) to identify significant ALFF changes.

### Statistical analysis

Clinical and demographic differences between MDD individuals and healthy controls were analysed using IBM SPSS Statistics version 27 (Armonk, NY, USA). Continuous variables were compared using independent-samples *t*-tests, while categorical variables were assessed using chi-square tests with a significance threshold of *P* < 0.05. To control for potential confounding factors, age, gender and head movement (quantified using mean framewise displacement) were included as covariates in the voxel-based analysis of ALFF differences between groups. This analysis was conducted in Statistical Parametric Mapping software (SPM12; Wellcome Trust Centre for Neuroimaging, University College London, UK; https://www.fil.ion.ucl.ac.uk/spm/). Group differences in ALFF were evaluated using two-sample *t*-tests, with statistical thresholds set to minimise type II errors and improve spatial specificity.^26^ Specifically, a voxel-level threshold of *P* < 0.001 was applied, followed by FWE correction at the cluster level and with a significance threshold of *P* < 0.05. Within each significant cluster, peak voxel coordinates (in MNI space) and associated *t*-values were reported to highlight the most robust group differences in ALFF. Correlation analyses between ALFF and clinical measures, including HAMD scores and illness duration, were performed using the simple regression function in SPM12.

## Results

**Fig. 1:**
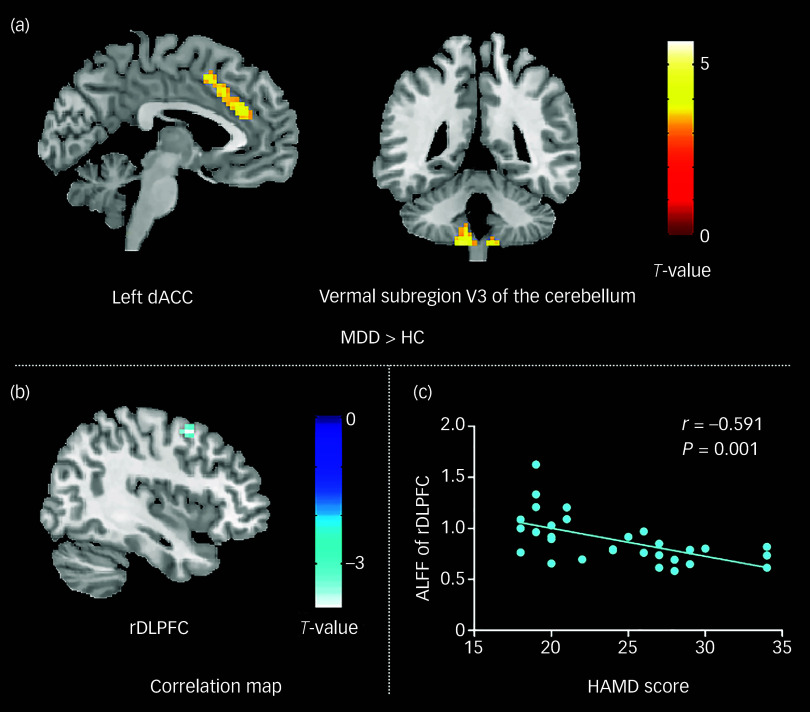
ALFF differences and correlation analyses in first-episode, drug-naive MDD patients. (a) Compared with healthy controls, individuals with MDD had higher ALFF in the bilateral cerebellum and left dACC. (b) Negative correlation between ALFF in the DLPFC and HAMD scores. (c) Scatter plot illustrating the significant negative relationship between ALFF in the right DLPFC and HAMD scores, with the regression line indicating the trend of association. At the individual voxel level, the statistical threshold was set at *P* < 0.001 and, at the cluster level, it was adjusted for multiple comparisons using family-wise error correction (*P* < 0.05). HAMD, Hamilton Rating Scale for Depression score; rDLPFC, right dorsolateral prefrontal cortex; dACC, dorsal anterior cingulate cortex; HC, healthy controls; ALFF, amplitude of low-frequency fluctuation; MDD, major depressive disorder.

Table 1 presents an overview of the clinical and demographic characteristics of all participants. There were no statistically significant differences in age (*P* = 0.754) or gender (*P* = 0.114) between those with MDD and healthy control groups. The results of the voxel-based ALFF analysis are summarised in Table 2 and Fig. 1(a). Compared with healthy controls, individuals with MDD exhibited significantly increased ALFF in the dACC and vermal subregion V3 of the cerebellum.^27^ All significant clusters survived FWE correction at *P* < 0.05, following a voxel-wise threshold of *P* < 0.001. No significant decreases in ALFF were observed in the MDD group.

**Table 1:** Demographic and clinical characteristics of all participants

Participant group	MDD	HC	*P*-value
	(*n* = 30)	(*n* = 52)	
Age, mean (s.d.), years	36.27 (12.05)	35.38 (12.31)	0.754
Gender, *n* (% male)	8 (26.7)	23 (44.2)	0.114
Education, mean (s.d.), years	12.33 (4.53)		
Illness duration, mean (s.d.), weeks	14.13 (16.39)		
HAMD score, mean (s.d.)	24.20 (5.08)		

MDD, major depressive disorder; HC, healthy controls; HAMD, Hamilton Rating Scale for Depression.

**Table 2:** Region-specific differences in amplitude of low-frequency fluctuations between first-episode, drug-naive patients with major depressive disorder and healthy controls

Brain region	Cluster size	Talairach coordinates (MNI)			*T*-value	*P*-value (FWE)
		*x*	*y*	*z*		
Cerebellum (vermal subregion V3)	158	−12	−48	−60	5.71	<0.05
		12	−51	−60	4.95	<0.05
		9	−42	−57	4.57	<0.05
Left dorsal anterior cingulate cortex	89	−6	36	21	4.31	<0.05
		−6	21	36	3.83	<0.05
		−9	24	27	3.77	<0.05

MNI, Montreal Neurological Institute; *T*-value, statistical value of peak voxel showing differences in amplitude of low-frequency fluctuations (ALFF) between patients with major depressive disorder and healthy controls who were not receiving therapy at the time of their first episode. The statistical threshold was established at *P* < 0.001 for individual voxels, and modified to *P* < 0.05 for clusters based on family-wise error (FWE) correction for multiple comparisons.

Following analysis of the correlation between MDD clinical measures and ALFF parameters, patients showed a negative association (*r* = −0.591, *P* < 0.001) between HAMD score and ALFF in the right DLPFC (Fig. 1(b) and (c)). There were no associations found between ALFF and length of illness.

## Discussion

This study investigated resting-state cerebral intrinsic activity in first-episode, drug-naive individuals with MDD who had a relatively short illness duration. The results revealed increased ALFF in the dACC and vermal subregion V3 of the cerebellum, suggesting dysregulated cerebral intrinsic activity in the early stages of MDD, independent of medication effects. Furthermore, a negative correlation was observed between ALFF in the right DLPFC and HAMD scores, indicating that altered intrinsic activity in the right DLPFC could serve as a potential marker of depression severity in MDD patients. This study provides novel insights into the neurobiological alterations underlying the early stages of MDD, contributing to a better understanding of the disorder’s pathophysiology and potential biomarkers for early diagnosis and intervention.

To the best of our knowledge, the ACC can be functionally and anatomically subdivided into an ‘affective subdivision’, which includes the rostral ACC and subgenual ACC, and a ‘cognitive subdivision’, which encompasses the dACC.^27^ The dACC serves as a critical hub within the cognitive control network, which is responsible for higher-order cognitive processes such as decision-making, working memory and conflict resolution.^28^ A recent meta-analysis of neuroimaging studies has shown that cognitive control tasks consistently engage the dACC.^29^ Additionally, meta-analytic findings on cognitive deficits in MDD have identified impaired executive function as a hallmark of first-episode MDD.^10^

In individuals with executive dysfunction, the dACC may require compensatory hyperactivation to maintain normal cognitive functions, such as decision-making and conflict resolution. In the present study, we observed increased ALFF in the dACC of first-episode, drug-naive MDD patients, suggesting aberrant intrinsic activity in this region during the early course of the disorder.

In comparison with healthy controls, first-episode, drug-naive MDD individuals showed greater ALFF activity in the dACC. Furthermore, Liu et al discovered that drug-naive MDD patients have higher ALFF activity in the dACC.^30^ However, a previous meta-analysis of eight ALFF studies demonstrated that ACC activity increase was detected in treated patients but not in drug-naive MDD patients.^10^ That meta-analysis included five original studies on drug-naive patients, and four of these studies failed to find ALFF activation differences in the ACC between MDD patients and healthy controls; the tiny sample size might have been the cause of this disparity,^16^ and lower field strength of the magnetic resonance scanner^7^ in those studies than ours. Besides, the discrepancies between the findings of the present study and those reported in meta-analyses may be attributed to the lack of detailed subregional analyses in the latter, as well as to differences in patient sample characteristics, particularly regarding illness duration. In comparison with the study by Liu et al,^30^ our study specifically focuses on first-episode, drug-naive MDD patients with a mean illness duration of only 14 weeks, allowing for a more precise investigation of early-stage neural alterations before disease progression occurs. Additionally, the use of a more stringent statistical threshold (*P* < 0.001 with FWE correction) enhances the robustness and reliability of our findings, minimising the likelihood of false-positive results. Moreover, the observed significant negative correlation between ALFF in the right DLPFC and HAMD scores suggests a potential biomarker for depression severity, which may contribute to early diagnosis and clinical monitoring of MDD.

Additionally, we discovered that individuals with MDD had considerably higher ALFF than controls in vermal subregion V3 of the cerebellum. The functional abnormalities of the cerebellum are a common manifestation of MDD.^31^ Several fMRI studies have reported significantly increased ALFF and regional homogeneity (ReHo) in the cerebellum, including vermal subregion V3, in MDD patients compared with healthy controls,^7,32^ findings that align with the results of our study. Additionally, a study on medication-naive MDD patients demonstrated enhanced cerebellar connectivity with the ACC during the resting state, further supporting the involvement of the cerebellum in the neuropathophysiology of MDD.^33^ The cerebellum receives projections from DLPFC, the medial frontal cortex, ACC, the parietal and superior temporal areas and the hypothalamus, largely via the thalamus relay, all of which are relevant to cognitive processing.^34^ Vermal area V3 of the cerebellum involves various domains of cognition such as language processing, memory and attention.^35^ Studies of cognitive deficits demonstrated that attention was a trait marker and reduced in first-episode MDD.^19^ Hence, the increased ALFF activity in vermal subregion V3 of the cerebellum could be interpreted as a result of counteracting or delaying the decline of attention. For those with reduced attention, the cerebral intrinsic activity of vermal subregion V3 of the cerebellum may need to be enhanced to maintain near-normal function. Our finding of higher ALFF activity in vermal subregion V3 of the cerebellum may be of significant clinical interest. Future studies combining structural and functional MRI are warranted to clarify the findings.

In this investigation, we discovered a negative correlation between HAMD scores and ALFF in the right DLPFC, suggesting that cerebral-intrinsic activity of the right DLPFC may be applied as an indicator of depression severity in first-episode, drug-naive MDD patients. Specifically, ALFF activity of the right DLPFC would decrease as depression symptoms worsen in individual with MDD. Because an important central component in cognitive control networks is involved in MDD and participation in maintaining and manipulating working memory, goal-directed action and attentional control,^36^ the DLPFC has also attracted attention in research into the pathophysiology of MDD. Several studies reported hypoactivation of the DLPFC in patients while performing cognitive tasks such as planning, word generation and working memory.^37–39^ Wang et al found that ALFF activity in the DLPFC was decreased in first-episode, drug-naive patients with MDD relative to controls,^16^ although this result was not found in our study. The observed discrepancies may be attributed to differences in depressive symptom severity among study populations. In the study of Wang et al, patients exhibited more severe depressive symptoms than those in our cohort, which may have contributed to the disparity in findings. Our research highlights the critical role of the DLPFC in the manifestation of depressive symptoms in MDD. Additionally, it is important to acknowledge that years of education have been identified as a significant factor influencing the onset and progression of MDD. While our study collected data on education years for MDD individuals, similar information was not available for healthy controls, precluding a direct statistical comparison. This represents a limitation of the current study, and future research should aim to systematically collect education-related data across all participant groups to fully assess its potential impact on MDD.

The findings of this study indicate that first-episode, drug-naive individuals with MDD exhibit significantly increased ALFF in vermal subregion V3 of the cerebellum and dACC. These results suggest that abnormal changes in cerebral intrinsic activity occur in the early stages of MDD. The practical implications of these findings are significant, because they highlight the potential for brain imaging assessments in the early stages of depression to identify potential cognitive dysfunctions, thereby providing a basis for early intervention and treatment. Additionally, this study provides new insights into the early neurobiology of depression, which may contribute to the development of novel diagnostic and therapeutic strategies. However, several limitations should be acknowledged. First, the small sample size may limit the generalisability and statistical power of the findings. Future research should include larger cohorts to enhance the reliability of the results. Second, the cross-sectional design precludes the ability to track dynamic changes in brain activity over time. Future studies should employ longitudinal designs to assess neurobiological alterations before and after treatment, or across different stages of the disorder. Third, physiological noise may affect the accuracy of rs-fMRI data. To mitigate potential confounding effects, future studies should incorporate simultaneous monitoring of heart rate and respiratory rate to improve data reliability. Moreover, this study primarily focused on intrinsic brain activity alterations in first-episode, drug-naive MDD individuals, using ALFF as the primary measurement index. Due to the study design and resource constraints, specific assessments of attentional changes were not included. This decision was based on the study’s primary objective: to explore the relationship between ALFF and clinical parameters such as depression severity and illness duration, rather than specific cognitive function changes. Additionally, measuremnt of attention would require additional behavioural assessments or cognitive tasks, which could increase study complexity and duration. However, future research should incorporate attentional assessments, particularly investigating the relationship between attention and cerebellar ALFF changes. Such investigations could provide a more comprehensive understanding of cognitive impairments in MDD individuals and potentially identify biomarkers for early diagnosis and intervention.

By addressing these limitations, future studies can further elucidate the neurobiological mechanisms of MDD and provide stronger empirical evidence to support clinical practice.

Our study identified the dACC and vermal subregion V3 of the cerebellum as being closely associated with drug-naive MDD individuals when they first experienced depression, implying a disturbance of cerebral intrinsic activity in the early course of MDD without the interference of medication. Given the critical role of these regions in cognitive function,^29,36^ it is speculated that early increase in cerebral intrinsic activity within these regions may help delay or mitigate the progression of cognitive impairment in MDD individuals during the early stages of the disorder. This finding has important clinical implications, particularly for the early screening and timely intervention of MDD. Additionally, our study suggests that altered ALFF in the right DLPFC may serve as a potential biomarker for evaluation of depression severity in first-episode, drug-naive MDD individuals. This finding provides new insights into the neurobiological mechanisms underlying the early course of depression, which may contribute to the development of more effective diagnostic and therapeutic strategies.

## Data Availability

The data-sets used and/or analysed during the current study are available from the corresponding author on reasonable request.

## References

[1] Mo Z-Y , Qin Z-Z , Ye J-J , Hu X-X , Wang R , Zhao Y-Y , et al. The long-term spatio-temporal trends in burden and attributable risk factors of major depressive disorder at global, regional and national levels during 1990–2019: a systematic analysis for GBD 2019. Epidemiol Psychiatr Sci 2024; 33: e28. 38764153 10.1017/S2045796024000295 PMC11362682

[2] Berk M , Köhler-Forsberg O , Turner M , Penninx B , Wrobel A , Firth J , et al. Comorbidity between major depressive disorder and physical diseases: a comprehensive review of epidemiology, mechanisms and management. World Psychiatry Off J World Psychiatr Assoc (WPA) 2023; 22: 366–87. 10.1002/wps.21110 PMC10503929 37713568

[3] Zang YF , He Y , Zhu CZ , Cao QJ , Sui MQ , Liang M , et al. Altered baseline brain activity in children with ADHD revealed by resting-state functional MRI. Brain Dev-JPN 2007; 29: 83–91. 10.1016/j.braindev.2006.07.002 16919409

[4] Mohamed MA , Yousem DM , Tekes A , Browner N , Calhoun VD. Correlation between the amplitude of cortical activation and reaction time: a functional MRI study. Am J Roentgenol 2004; 183: 759–65. 15333368 10.2214/ajr.183.3.1830759

[5] Kublbock M , Woletz M , Hoflich A , Sladky R , Kranz GS , Hoffmann A , et al. Stability of low-frequency fluctuation amplitudes in prolonged resting-state fMRI. NeuroImage 2014; 103: 249–57. 25251869 10.1016/j.neuroimage.2014.09.038

[6] Zuo XN , Xing XX. Test-retest reliabilities of resting-state FMRI measurements in human brain functional connectomics: a systems neuroscience perspective. Neurosci Biobehav Rev 2014; 45: 100–18. 24875392 10.1016/j.neubiorev.2014.05.009

[7] Guo WB , Liu F , Xue ZM , Xu XJ , Wu RR , Ma CQ , et al. Alterations of the amplitude of low-frequency fluctuations in treatment-resistant and treatment-response depression: a resting-state fMRI study. Progr Neuropsychopharmacol Biol Psychiatry 2012; 37: 153–60. 10.1016/j.pnpbp.2012.01.011 22306865

[8] Fan T , Wu X , Yao L , Dong J. Abnormal baseline brain activity in suicidal and non-suicidal patients with major depressive disorder. Neurosci Lett 2013; 534: 35–40. 23201633 10.1016/j.neulet.2012.11.032

[9] Jing B , Liu CH , Ma X , Yan HG , Zhuo ZZ , Zhang Y , et al. Difference in amplitude of low-frequency fluctuation between currently depressed and remitted females with major depressive disorder. Brain Res 2013; 1540: 74–83. 24121137 10.1016/j.brainres.2013.09.039

[10] Zhou M , Hu X , Lu L , Zhang L , Chen L , Gong Q , et al. Intrinsic cerebral activity at resting state in adults with major depressive disorder: a meta-analysis. Progr Neuropsychopharmacol Biol Psychiatry 2017; 75: 157–64. 10.1016/j.pnpbp.2017.02.001 28174129

[11] Zhang XC , Zhu XL , Wang X , Zhu XZ , Zhong MT , Yi JY , et al. First-episode medication-naive major depressive disorder is associated with altered resting brain function in the affective network. Plos ONE 2014; 9: e85241. 24416367 10.1371/journal.pone.0085241 PMC3887023

[12] Delaveau P , Jabourian M , Lemogne C , Guionnet S , Bergouignan L , Fossati P. Brain effects of antidepressants in major depression: a meta-analysis of emotional processing studies. J Affect Disord 2011; 130: 66–74. 21030092 10.1016/j.jad.2010.09.032

[13] Fales CL , Barch DM , Rundle MM , Mintun MA , Mathews J , Snyder AZ , et al. Antidepressant treatment normalizes hypoactivity in dorsolateral prefrontal cortex during emotional interference processing in major depression. J Affect Disord 2009; 112: 206–11. 18559283 10.1016/j.jad.2008.04.027 PMC2825146

[14] Cheng Y , Xu J , Arnone D , Nie B , Yu H , Jiang H , et al. Resting-state brain alteration after a single dose of SSRI administration predicts 8-week remission of patients with major depressive disorder. Psychol Med 2017; 47: 438–50. 27697079 10.1017/S0033291716002440

[15] Guo W , Liu F , Yu M , Zhang J , Zhang Z , Liu J , et al. Functional and anatomical brain deficits in drug-naive major depressive disorder. Progr Neuropsychopharmacol Biol Psychiatry 2014; 54: 1–6. 10.1016/j.pnpbp.2014.05.008 24863419

[16] Wang L , Dai W , Su Y , Wang G , Tan Y , Jin Z , et al. Amplitude of low-frequency oscillations in first-episode, treatment-naive patients with major depressive disorder: a resting-state functional MRI study. Plos ONE 2012; 7: e48658. 23119084 10.1371/journal.pone.0048658 PMC3485382

[17] Gao Q , Zou K , He Z , Sun X , Chen H. Causal connectivity alterations of cortical-subcortical circuit anchored on reduced hemodynamic response brain regions in first-episode drug-naive major depressive disorder. Sci Rep 2016; 6: 21861. 26911651 10.1038/srep21861 PMC4766513

[18] Liu J , Ren L , Womer FY , Wang J , Fan G , Jiang W , et al. Alterations in amplitude of low frequency fluctuation in treatment-naïve major depressive disorder measured with resting-state fMRI. Hum Brain Mapp 2014; 35: 4979–88. 24740815 10.1002/hbm.22526 PMC6869357

[19] Lui S , Wu Q , Qiu L , Yang X , Kuang W , Chan RC , et al. Resting-state functional connectivity in treatment-resistant depression. Am J Psychiatry 2011; 168: 642–8. 21362744 10.1176/appi.ajp.2010.10101419

[20] Williams JB. A structured interview guide for the Hamilton Depression Rating Scale. Arch Gen Psychiatry 1988; 45: 742–7. 3395203 10.1001/archpsyc.1988.01800320058007

[21] Chao-Gan Y , Yu-Feng Z. DPARSF: a MATLAB toolbox for ‘Pipeline’ data analysis of resting-state fMRI. Front Syst Neurosci 2010; 4: 13. 20577591 10.3389/fnsys.2010.00013 PMC2889691

[22] Lui S , Li T , Deng W , Jiang L , Wu Q , Tang H , et al. Short-term effects of antipsychotic treatment on cerebral function in drug-naive first-episode schizophrenia revealed by ‘resting state’ functional magnetic resonance imaging. Arch Gen Psychiatry 2010; 67: 783–92. 20679586 10.1001/archgenpsychiatry.2010.84

[23] Biswal B , Yetkin FZ , Haughton VM , Hyde JS. Functional connectivity in the motor cortex of resting human brain using echo-planar MRI. Magn Reson Med 1995; 34: 537–41. 8524021 10.1002/mrm.1910340409

[24] Raichle ME , MacLeod AM , Snyder AZ , Powers WJ , Gusnard DA , Shulman GL. A default mode of brain function. Proc Natl Acad Sci USA 2001; 98: 676–82. 11209064 10.1073/pnas.98.2.676 PMC14647

[25] Woo CW , Krishnan A , Wager TD. Cluster-extent based thresholding in fMRI analyses: pitfalls and recommendations. NeuroImage 2014; 91: 412–9. 24412399 10.1016/j.neuroimage.2013.12.058 PMC4214144

[26] Monkul ES , Hatch JP , Sassi RB , Axelson D , Brambilla P , Nicoletti MA , et al. MRI study of the cerebellum in young bipolar patients. Prog Neuropsychopharmacol Biol Psychiatry 2008; 32: 613–9. 18272276 10.1016/j.pnpbp.2007.09.016 PMC2778760

[27] Pizzagalli DA. Frontocingulate dysfunction in depression: toward biomarkers of treatment response. Neuropsychopharmacology 2011; 36: 183–206. 20861828 10.1038/npp.2010.166 PMC3036952

[28] Dutta A , McKie S , Deakin JF. Resting state networks in major depressive disorder. Psychiat Res 2014; 224: 139–51. 10.1016/j.pscychresns.2014.10.003 25456520

[29] Niendam TA , Laird AR , Ray KL , Dean YM , Glahn DC , Carter CS. Meta-analytic evidence for a superordinate cognitive control network subserving diverse executive functions. Cogn Affect Behav Neurosci 2012; 12: 241–68. 22282036 10.3758/s13415-011-0083-5 PMC3660731

[30] Liu J , Ren L , Womer FY , Wang J , Fan G , Jiang W , et al. Alterations in amplitude of low frequency fluctuation in treatment-naive major depressive disorder measured with resting-state fMRI. Hum Brain Mapp 2014; 35: 4979–88. 24740815 10.1002/hbm.22526 PMC6869357

[31] Phillips JR , Hewedi DH , Eissa AM , Moustafa AA. The cerebellum and psychiatric disorders. Front Public Health 2015; 3: 66. 26000269 10.3389/fpubh.2015.00066 PMC4419550

[32] Guo WB , Sun XL , Liu L , Xu Q , Wu RR , Liu ZN , et al. Disrupted regional homogeneity in treatment-resistant depression: a resting-state fMRI study. Prog Neuropsychopharmacol Biol Psychiatry 2011; 35: 1297–302. 21338650 10.1016/j.pnpbp.2011.02.006

[33] Ma Q , Zeng LL , Shen H , Liu L , Hu D. Altered cerebellar-cerebral resting-state functional connectivity reliably identifies major depressive disorder. Brain Res 2013; 1495: 86–94. 23228724 10.1016/j.brainres.2012.12.002

[34] Rapoport M , van Reekum R , Mayberg H. The role of the cerebellum in cognition and behavior: a selective review. J Neuropsychiat Clin Neurosci 2000; 12: 193–8. 10.1176/jnp.12.2.193 11001597

[35] Park MTM , Pipitone J , Baer LH , Winterburn JL , Shah Y , Chavez S , et al. Derivation of high-resolution MRI atlases of the human cerebellum at 3 T and segmentation using multiple automatically generated templates. Neuroimage 2014; 95: 217–31. 24657354 10.1016/j.neuroimage.2014.03.037

[36] Miller EK , Cohen JD. An integrative theory of prefrontal cortex function. Annu Rev Neurosci 2001; 24: 167–202. 11283309 10.1146/annurev.neuro.24.1.167

[37] Fincham JM , Carter CS , van Veen V , Stenger VA , Anderson JR , Neural mechanisms of planning: a computational analysis using event-related fMRI, Proc Natl Acad Sci USA 2002; 99: 3346–51. 11880658 10.1073/pnas.052703399 PMC122521

[38] Okada G , Okamoto Y , Morinobu S , Yamawaki S , Yokota N. Attenuated left prefrontal activation during a verbal fluency task in patients with depression. Neuropsychobiology 2003; 47: 21–6. 12606841 10.1159/000068871

[39] Rajah MN , D’Esposito M. Region-specific changes in prefrontal function with age: a review of PET and fMRI studies on working and episodic memory. Brain J Neurol 2005; 128: 1964–83. 10.1093/brain/awh608 16049041

